# Leadership program with skills training for general practitioners was highly accepted without improving job satisfaction: the cluster randomized IMPROVE*job* study

**DOI:** 10.1038/s41598-022-22357-z

**Published:** 2022-10-25

**Authors:** Lukas Degen, Julian Göbel, Karen Minder, Tanja Seifried-Dübon, Brigitte Werners, Matthias Grot, Esther Rind, Claudia Pieper, Anna-Lisa Eilerts, Verena Schröder, Achim Siegel, Anika Hüsing, Karl-Heinz Jöckel, Monika A. Rieger, IMPROVEjob Research Cooperation, B. Weltermann, K. Minder, L. Degen, J. Göbel, M. Schmidt, A. Dreher, S. Kasten, F. Junne, T. Seifried-Dübon, F. Stuber, S. Zipfel, B. Werners, M. Grot, L. Imhoff, J. Block, M. Rieger, E. Rind, A. Wagner, E. Tsarouha, S. Burgess, A. Siegel, K. H. Jöckel, C. Pieper, V. Schröder, M. Brinkmann, A. L. Eilerts, Birgitta M. Weltermann

**Affiliations:** 1 Institute of General Practice and Family Medicine, University Hospital Bonn, Venusberg-Campus 1, 53127 Bonn, Germany; 2 Department of Psychosomatic Medicine and Psychotherapy, University Hospital Tuebingen, Osianderstraße 5, 72076 Tübingen, Germany; 3 Institute of Management, Operations Research, Ruhr University Bochum, Universitätsstr. 150, 44801 Bochum, Germany; 4 Institute of Occupational and Social Medicine and Health Services Research, University Hospital Tuebingen, Wilhelmstr. 27, 72074 Tübingen, Germany; 5 Institute for Medical Informatics, Biometry and Epidemiology, University Hospital Essen, University of Duisburg-Essen, Hufelandstr. 55, 45147 Essen, Germany; 6 Center for Clinical Trials, University Hospital Essen, University of Duisburg-Essen, Hufelandstr. 55, 45147 Essen, Germany

**Keywords:** Occupational health, Epidemiology

## Abstract

Leadership has become an increasingly important issue in medicine as leadership skills, job satisfaction and patient outcomes correlate positively. Various leadership training and physician psychological well-being programmes have been developed internationally, yet no standard is established in primary care. The IMPROVE*job* leadership program was developed to improve job satisfaction among German general practitioners and practice personnel. Its acceptance and effectiveness were evaluated. The IMPROVE*job* intervention is a participatory, interdisciplinary and multimodal leadership intervention that targets leadership, workflows and communication in general practices using three elements: (1) two leadership workshops with skills training; (2) a toolbox with printed and online material, and (3) a 9-month implementation phase supported by facilitators. A cluster-randomised trial with a waiting-list control evaluated the effectiveness on the primary outcome job satisfaction assessed by the Copenhagen Psychosocial Questionnaire (range 0–100). A mixed-methods approach with questionnaires and participant interviews evaluated the acceptance of the intervention and factors influencing the implementation of intervention content. Statistical analyses respected the clustered data structure. The COVID-19 pandemic necessitated intervention adjustments: online instead of on-site workshops, online material instead of facilitator practice visits. Overall, 52 of 60 practices completed the study, with altogether 70 practice leaders, 16 employed physicians, and 182 practice assistants. According to an intention-to-treat analysis, job satisfaction decreased between baseline and follow-up (not significantly) in the total study population and in both study arms, while the subgroup of practice leaders showed a non-significant increase. A mixed multilevel regression model showed no effect of the intervention on job satisfaction (*b* = − 0.36, *p* > 0.86), which was influenced significantly by a greater sense of community (*b* = 0.14, *p* < 0.05). The acceptance of the IMPROVE*job* workshops was high, especially among practice leaders compared to assistants (1 = best to 5 = worst): skills training 1.78 vs. 2.46, discussions within the practice team 1.87 vs. 2.28, group discussions 1.96 vs. 2.21. The process evaluation revealed that the COVID-19 pandemic complicated change processes and delayed the implementation of intervention content in practice routines. The workshops within the participatory IMPROVE*job* intervention were rated very positively but the multimodal intervention did not improve job satisfaction 9 months into the pandemic. Qualitative data showed an impairment of implementation processes by the unforeseeable COVID pandemic.

**Trial registration** Registration number: DRKS00012677 on 16/10/2019.

## Introduction

In the last decades, leadership has become an increasingly important topic in medicine, with the need to especially train physician leaders^1, 2^. In graduate education, the ‘physician as leader’ is conceptualized in the CanMEDS roles, but no standard for leadership training in primary care and other specialties has been developed^3, 4^. Four reviews comparing various leadership programs showed considerable diversity regarding target groups (physicians of various experience levels), specialty focus (primary care and other fields), program aims (e.g., clinical leadership for integrated primary care), theoretical foundation and methodological approaches (scoping, narrative vs. systematic reviews)^4–7^.

All reviews mentioned describe leadership as a dynamic process between persons that is oriented towards individual, group or organizational goals and is associated with influence^4^. Previous leadership programs drew on different theories, e.g., transformational and transactional leadership as frequently used modern concepts^4–7^. Transactional leadership is based on a mutual exchange between leader and employees (e.g., rewarding previously negotiated objectives)^8^, while transformational leadership addresses the leader's promotion of intrinsic motivation and communication of vision^9^. Both theories are well-studied, established and complement each other theoretically and in practice^4^. Methodologically, the reviewed interventions combine various learning methods, e.g., seminars, lectures, group work, mentoring, multi-source and action-based feedback. A 2014 review by Frich et al. identified 12 programs which involved the use of simulation exercises (simulated practice and/or role-play)^6^. Of these, six interventions improved outcomes on the system level, e.g., staff-reported quality of care, participant career success, improvements of disease management programs, and customer satisfaction^6^. Drawing on various occupational fields, a meta-analysis by Judge and Piccolo showed that employees’ job satisfaction correlates positively with transformational leadership (ρ = 0.58) and contingent reward leadership as a dimension of transactional leadership (ρ = 0.64)^10^. However, the authors of many reviews agree, that more rigorous research on leadership and leadership training measured by relevant subjective and objective outcomes is needed^4–7^.

A study with more than 200,000 German professionals from the hospital setting and other occupational fields highlighted the importance of leadership as the most important predictor of job satisfaction^11^, which in turn was deeply linked to work-related factors such as workload, team support, recognition, bureaucracy, and income in European general practitioner (GP) populations^12, 13^. Also, job satisfaction was associated with emotional exhaustion and stress related to patient care^14^. Interventions to optimize job satisfaction showed mixed results. A 6-month professional coaching of 88 physicians, including family physicians, improved quality of life and resilience while reducing emotional exhaustion and burnout rates, yet job satisfaction did not change^15^. Job satisfaction among 45 Spanish GPs improved after participating in a multimodal training program with an integrated systemic therapy approach^16^. While job satisfaction was widely studied in GP populations, intervention studies addressing leadership and job satisfaction in this setting are missing.

The IMPROVE*job* intervention conceptualized a participatory, interdisciplinary and multimodal leadership program for GPs to improve job satisfaction. It drew on the transformational and transactional leadership theories as well as the leader member exchange theory^17^. Using innovative skills trainings, the intervention aimed at practice-relevant leadership skills^18, 19^. The effectiveness of the IMPROVE*job* leadership program on job satisfaction of GP practice leaders and practice personnel and its acceptance were evaluated in a cluster-randomized controlled trial.

## Methods

### Study design, sample size and randomisation

**Figure 1:**
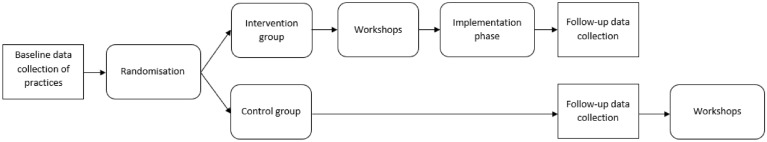
Study design^18^.

The IMPROVE*job* study evaluated the effectiveness of the IMPROVE*job* intervention on job satisfaction among practice leaders and practice personnel. It was conducted as a cluster-randomised controlled trial (cRCT) with a waiting-list control group, i.e., control group participants received the intervention after follow-up data collection (see Fig. 1). After baseline data collection, the practices were randomised to the two study arms with the intervention group receiving the intervention lasting 9 months. All participating practices were recruited in the Greater Bonn/Cologne region of North-Rhine Westphalia, Germany. According to the sample size calculation, we targeted a total of 56 practices with an average of 4 participants per practice for recruitment, allowing for 2 dropouts in each study arm (for details see^18^). The randomisation was carried out by the Centre for Clinical Trials Essen after baseline data collection. The randomisation was stratified for (a) single versus group practice and (b) teaching versus non-teaching practice.

### Inclusion and exclusion criteria

We included practices if the practice leader was registered as a general practitioner of the Association of Statutory Health Insurance Physicians of North-Rhine and/or belonged to the teaching physician network of the University of Bonn or Cologne. We excluded practices if they were in extraordinary situations such as an upcoming retirement of the leader. In addition, we excluded any practices that had participated in the development of the IMPROVEjob intervention or the feasibility study of the intervention.

### Informed consent, data collection and outcome measures

All participants provided written informed consent. Data collection took place before randomisation and 9 months after the intervention.

The primary outcome of the IMPROVE*job* study was a change in job satisfaction, measured with the German version of the Copenhagen Psychosocial Questionnaire (German COPSOQ, Version 2018). The respective job satisfaction scale combines five items and an additional global item (‘How pleased are you with your job as a whole, everything taken into consideration?’) using a 5-point Likert scale and were transformed to a score ranging from 0 (‘not satisfied at all’) to 100 (‘fully satisfied’) based on the COPSOQ guidelines^20^.

The questionnaire comprised various secondary outcomes which are detailed in the published study protocol^18^. Of these, we used the following measurements for the analyses presented here: COPSOQ scales ‘social support’ (B8: 1–4) and ‘sense of community’ (B8: 8–9). The scores for each dimension were transformed as recommended, ranging from 0 (minimum value, ‘do not agree at all’) to 100 (maximum value, ‘fully agree’)^20, 21^. Leadership was assessed using the questionnaire on Integrative Leadership (FIF, Fragebogen zur Integrativen Führung)^22^. We used the six dimensions of transformational leadership (fostering innovation, team spirit development, performance development, individuality focus, providing a vision, being a role model) and the two dimensions of transactional leadership (goal setting, management by exception)^22, 23^. The workshops and the specific contents of the intervention were assessed at follow-up using an adapted scale based on the German school grading system (1 = best to 5 = worst).

### Process analysis by qualitative interviews addressing factors influencing implementation

After the 9-month implementation phase, semi-structured qualitative interviews were conducted with four practice leaders and three practice assistants from the intervention group by phone (n = 4) and face-to-face (n = 3). The interviews were transcribed and analysed by qualitative content analysis^24^. The interview guide addressed the following topics: planned and actual changes in the practices after workshop participation, facilitators and barriers to change processes and experiences with the IMPROVE*job* facilitators.

### Intervention

**Figure 2:**
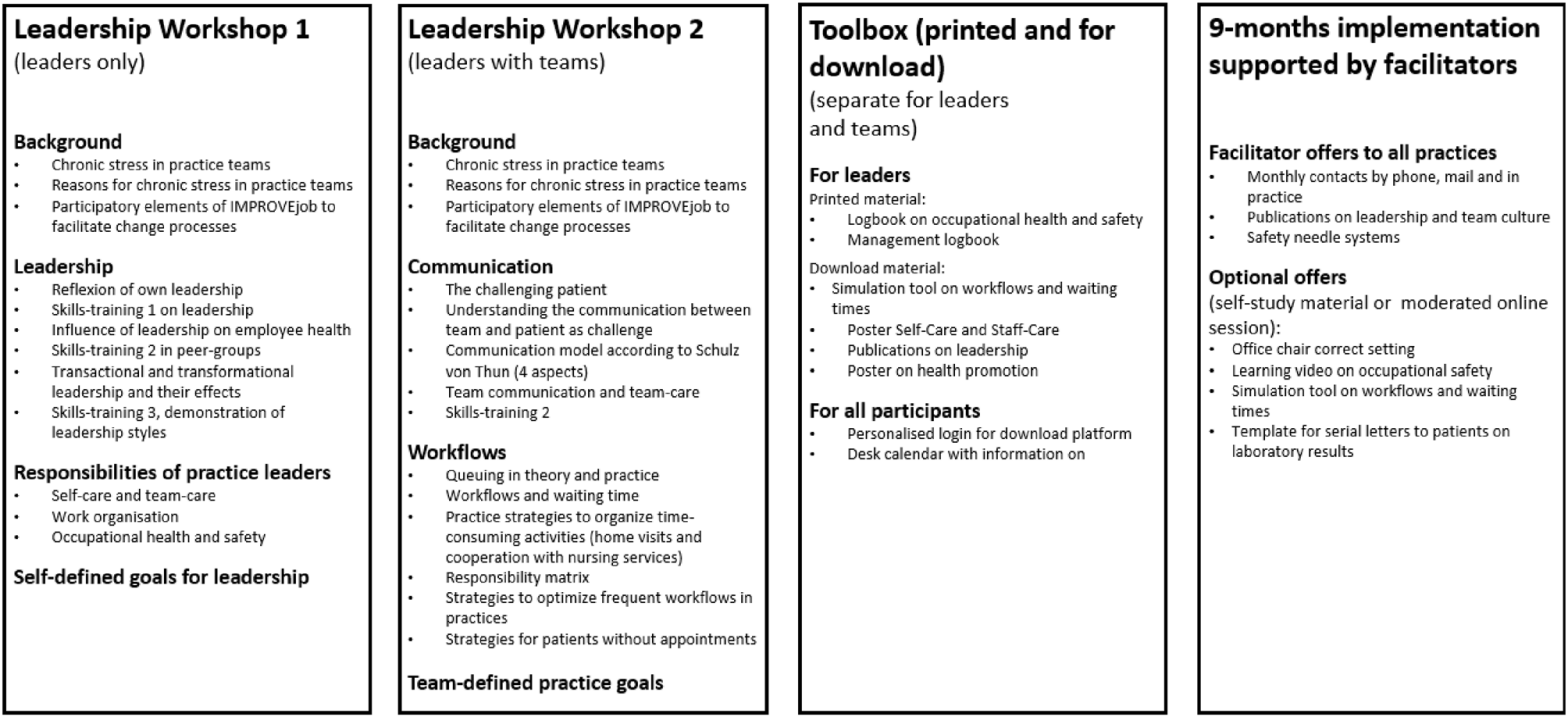
Elements of the IMPROVE*job* intervention^[(25, p. 5)]^.

The IMPROVE*job* intervention consisted of three core elements (see Fig. 2):

1. Two IMPROVE*job* leadership workshops (3.5 h each): one for practice leaders (practice leaders and physicians with leadership responsibilities) and one for the practice leaders and their teams,
2. The IMPROVE*job* toolbox with additional materials, and
3. The 9-month implementation phase supported by IMPROVE*job* facilitators.

The two intervention workshops for each practice took place between November 2019 and August 2020 and were conducted with an interval of 2 weeks. Depending on availability and practice size, a total of 3 to 6 practice teams took part in each workshop.

The workshops were led by one of two academic primary care physicians and included presentations by the researchers from the various fields in addition to interactive elements, self-reflection, peer exchange, and several leadership skills training sessions supported by simulation patients. All skills training sessions were based on a fictional scenario confronting the participants with situations challenging their individual leadership skills. Leadership workshop 1 for physician leaders and physicians with leadership responsibilities addressed the topics ‘role of the executive’, ‘leadership styles’ and ‘occupational health and safety for GP practices’ in theory and practice. The first skills training sessions in leadership workshop 1 addressed the scenario of a leader confronted with a conflict between practice team members. The second skills training was a presentation by the research team on a fictitious team session to illustrate various aspects of transformational and transactional leadership.

Leadership workshop 2 for physicians with leadership responsibilities and their practice teams concentrated on ‘work organisation including appointment scheduling’, ‘workplace health promotion’ and ‘communication with patients’. Further skills training sessions (two for the practice assistants, one for the practice leaders) addressed communication with challenging patients. In addition, this workshop focussed on the practice team to analyse common workflows and integrate optimized procedures into the practice workflow.

The IMPROVE*job* toolbox comprised printed and online material which was introduced in the workshops: The ‘management logbook’ for physicians with leadership responsibilities, the ‘employee logbook’, the desk calendar for practice teams and additional material for downloading.

The 9-month implementation phase, supported by IMPROVE*job* facilitators, began after leadership workshop 2. The two facilitators were trained practice assistants with profound professional experience who assisted the practices during the change processes. The facilitators’ main tasks were to remind the practice of the IMPROVE*job* study, the self-defined practice goal, and to offer additional toolbox material.

### Changes of the study protocol and study conduct due to the COVID-19 pandemic

The first lockdown in the COVID-19 pandemic started in March 2020 during the intervention phase and required the following changes:

1. Online instead of on-site workshops: Except for one, all workshops of the intervention group were conducted on-site prior to the pandemic. The remaining on-site workshop was split into five online sessions due to organizational reasons. All control group workshops were shifted to the online format.
2. Adaptation of workshop content for the online format: The workshop duration was reduced from 3.5 to 2 h with some educational material being shifted to the toolbox. The skills training sessions with simulation patients were continued but modified to allow for an online format.
3. Written and online offers instead of practice visits in the implementation phase: Due to contact restrictions, facilitators were unable to perform practice visits. Practices received monthly facsimiles with educational material, phone calls and offers for videos and/or online sessions on various topics.
4. For n = 11 busy practices that were unable to complete the follow-up questionnaire, a one-page option covering only the main outcome job satisfaction was offered.

### Statistical analysis and ethics statement

We used standard statistics for a multilevel description of the sample and the various items respecting the clustered data structure. Following our study protocol, we calculated all standardized scales following the recommendations of the respective scales^18^. Multilevel regression analyses were performed to compare the change in job satisfaction between baseline and follow-up in the intervention and the control group (primary outcome). In addition, according to results from recent literature^11^ we analysed for associations between the change in job satisfaction assessment (difference between baseline and follow-up) and sociodemographic data (age, gender, occupational group and working full-time) as well as the secondary outcomes transformational and transactional leadership scores, social support and sense of community at follow-up. All regression analyses respected the clustered data structure. The additional evaluation of the intervention elements used a 5-point Likert scale linked to the German school grading system (1 = best/very satisfied to 5 = worst/very unsatisfied). SPSS Statistics 27 (IBM Corporation, 2020), SAS 9.4 and RStudio were used for statistical analyses. The significance level was set at p < 0.05. Results are reported according to the CONSORT 2010 checklist of information to include when reporting a randomised trial (see Additional File 1).

The study was first approved by the Ethics Committee of the Medical Faculty of the University of Bonn (reference number: 057/19, date of approval: 20 February 2019).

### Ethics approval and consent to participate

The study complies with the ethical principles of the World Medical Association Declaration of Helsinki. The study was first approved by the Ethics Committee of the Medical Faculty of the University of Bonn (reference number: 057/19, date of approval: 20/02/2019). In addition, the Ethics Committees of the Medical Association of North-Rhine (ref. no.: 2019107), and of the Medical Faculty, University Hospital of Tuebingen (Project No.: 446/2019BO2) approved the study protocol. All participants provided written informed consent before participating in the study.

## Results

A total of 52 practices with 268 participants (intervention group = 129, control group = 139) completed the study: 70 practice leaders, 16 employed physicians and 182 practice assistants. The drop-out comprised 8 practices (*n* = 98 participants) with 14 practice leaders, 12 employed physicians and 72 practice assistants, *n* = 53 of whom from the intervention group and n = 45 from the control group. There were no statistically significant differences for gender, working full-time, job satisfaction and chronic stress at baseline between individuals with and without a follow-up, while the mean age differed (45.5 [with follow-up] vs. 41.4 years [only baseline]). At follow-up, 23 participants from 11 practices completed the short questionnaire (8.6%): 12 practice leaders, 1 employed physician and 10 practice assistants.

The leaders more frequently worked full-time and had been in their current practice for longer. About half of the leaders were female (51.4%), as were all practice assistants. Of the non-physician personnel, 83.4% were certified practice assistants, while 7.1% were still in training (see Table 1).

**Table 1:** Sociodemographic characteristics of the participants who completed follow-up (n = 268).

Variable	Total sample	Practice leader	Employed physician	Practice assistant
	N = 268	N = 70	N = 16	N = 182
Female, %	85.4	51.4	68.8	100.0
Age in years, mean (SD)	45.5 (12.3)	53.6 (5.9)	47.2 (9.9)	42.2 (12.8)
Years in current practice, mean (SD)	12.5 (9.2)	16.56 (8.1)	8.4 (6.9)	11.12 (9.2)
Working full-time, %	54.8	90.0	37.5	42.3
Living in a relationship/married, %	81.5	88.2	93.8	77.9
Persons in household over 18 years, mean (SD)	2.1 (0.9)	2.09 (0.9)	2.07 (0.5)	2.15 (0.9)
Persons in household under 18 years, mean (SD)	1.1 (1.0)	1.2 (1.2)	1.2 (1.1)	1.1 (0.9)
Care for next-of-kin, %	27.4	35.8	15.4	25.7
**Professional characteristics of physicians (N = 86)**				
Years since accreditation as physician, mean (SD)	25.6 (8.3)	27.1 (8.1)	19.4 (10.4)	–
Physician in GP training, %			37.5	
**Number of patients in 3 months, %**				
< 750	25.0	22.9	35.7	
751–1000	28.6	28.6	28.6	
1001–1250	23.8	22.9	28.6	
> 1250	22.6	25.7	7.1	
**Professional characteristics of practice assistants (N = 182)**				
Years since graduation, mean (SD)				22.0 (13.5)
Qualification as practice assistant, %				83.4
Practice assistant in training, %				7.1
Average working hours in last 3 months per week, mean (SD)				31.0 (89.0)

As detailed in Table 2, the mean job satisfaction of the practice leaders increased from baseline to follow-up, while it decreased among practice assistants.

**Table 2:** Intention-to-treat analysis: multilevel regression analyses for the primary outcome job satisfaction for total sample and by professional groups (stratified by study arm) (n = 268).

	Study arm	Baseline Mean (95% CI)	Follow-up Mean (95% CI)	Change from baseline to follow-up Mean (95% CI)
Total study population	Intervention (n = 129)	73.41 (70.24 to 76.58)	71.95 (68.07 to 75.83)	− 1.31 (− 4.13 to 1.50)
	Control (n = 139)	75.19 (72.00 to 78.39)	74.06 (70.10 to 78.02)	− 0.96 (− 3.77 to 1.85)
**Subpopulations**				
Practice leader	Intervention (n = 37)	74.37 (69.24 to 79.51)	79.46 (75.45 to 83.46)	5.78 (0.86 to 10.70)
	Control (n = 33)	79.85 (74.40 to 85.31)	83.08 (78.79 to 87.37)	359 (− 1.62 to 8.80)
Employed physician*	Intervention (n = 5)	79.17 (68.78 to 89.55)	74.17 (59.06 to 89.27)	− 5.00 (− 20.3 to 10.27)
	Control (n = 11)	77.65 (70.65 to 84.65)	80.23 (70.04 to 90.41)	2.87 (− 7.92 to 13.67)
Practice assistant	Intervention (n = 87)	72.65 (68.55 to 76.74)	68.43 (63.58 to 73.28)	− 4.01 (− 7.31 to − 0.71)
	Control (n = 95)	73.44 (69.35 to 77.53)	70.36 (65.49 to 75.24)	− 2.97 (− 6.22 to 0.29)

*Low case number; model fit does not converge. Values are reported without cluster adjustment.

In the intention-to-treat analysis for the primary outcome, the multilevel regression model estimated an effect size of − 0.36 (CI 95%: − 4.34 to 3.62; p = 0.86).

In a multilevel regression model, age (*t* = 3.78, *b* = 0.28) and sense of community at follow-up (*t* = 2.67, *b* = 0.14) were found to significantly influence the change in job satisfaction between baseline and follow-up, while the study arm and the other variables had no significant influence. For details see Table 3.

**Table 3:** Mixed model on the difference in job satisfaction between baseline and follow-up (model 1).

	Difference in job satisfaction between baseline and follow-up		
	b	SEb	t
Age	0.28	0.07	3.78***
Sex	− 2.22	2.83	− 0.79
Working time	− 2.25	1.83	− 1.23
Practice owner	− 1.15	3.05	− 0.38
Employed physician	− 0.33	3.26	− 0.10
Social support	− 0.01	0.05	− 0.23
Sense of community	0.14	0.05	2.67**
Transformational leadership	2.19	1.42	1.54
Transactional leadership	1.38	1.31	1.06
Intervention	1.91	1.67	1.15

*p < 0.05, **p < 0.01, ***p < 0.001, *b* regression coefficient b, *SEB* standard error, *t* t-value, a coded as 0 = male, 1 = female, b coded as 1 = yes, 0 = no. Significant values are in bold.

### Evaluation of the workshops and workshop contents

**Figure 3:**
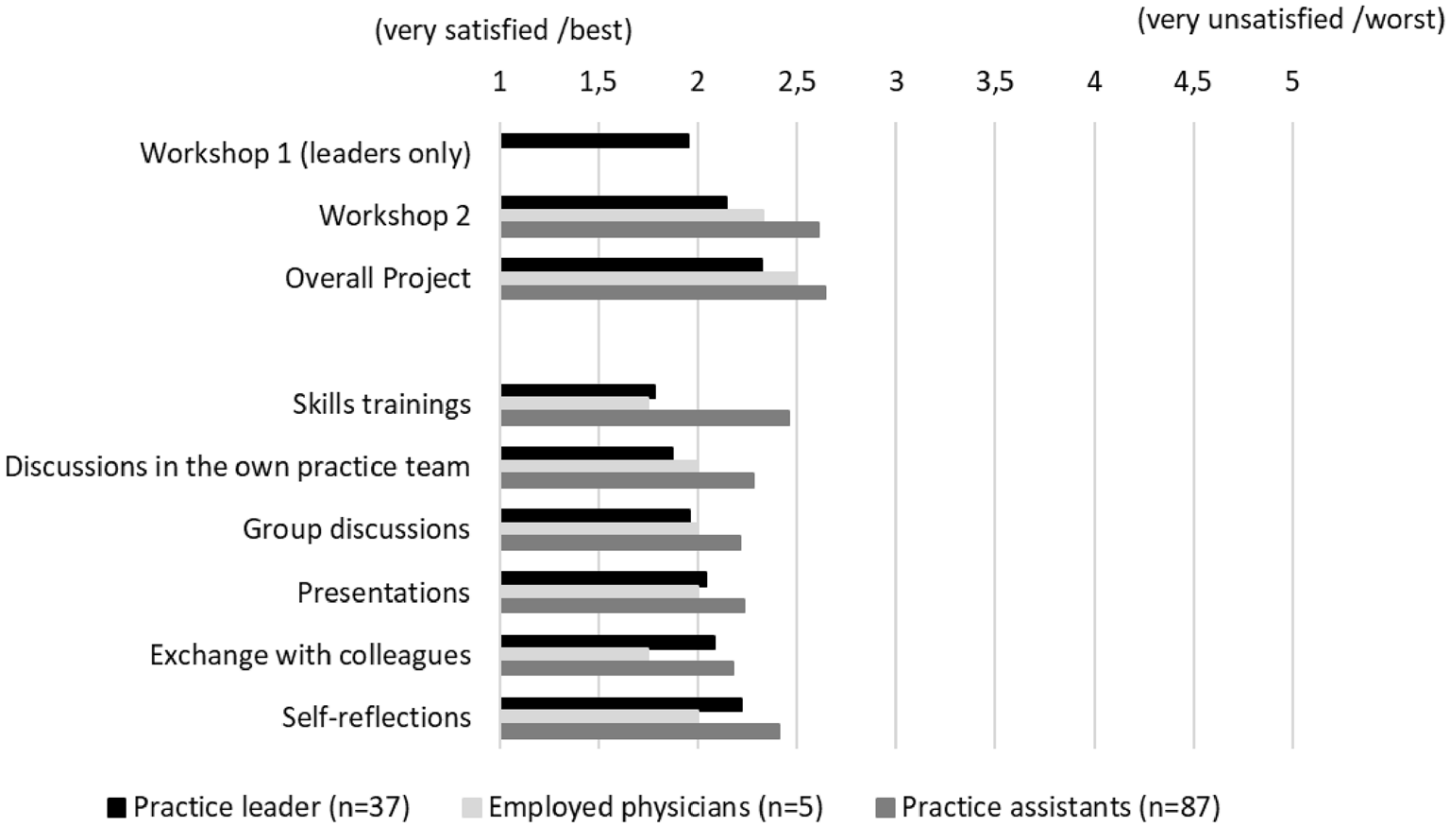
Evaluation of the intervention elements.

The workshops were rated by 25 of 37 (67.6%) practice leaders, 4 of 5 (80%) employed physicians and 69 of 87 (79.3%) practice assistants. The evaluation of the workshops, performed on an individual level, showed that the workshops were rated well. The highest ratings were given by physician leaders: skills training (mean 1.78), group discussions (mean 1.96), and discussions within their own practice team (mean 1.87) (for details see Table 4 and Fig. 3).

**Table 4:** Evaluation of the workshop elements by the intervention group at follow-up (total and stratified by profession) using a five-point scale (1 = very satisfied/best to 5 = very unsatisfied/worst).

	Total sample (n = 129)		Practice leader (n = 37)		Employed physicians (n = 5)		Practice assistants (n = 87)	
	N	Mean (SD)	N	Mean (SD)	N	Mean (SD)	N	Mean (SD)
Skills trainings	84	2.24 (0.79)	23	1.78 (0.80)	4	1.75 (0.96)	57	2.46 (0.68)
Discussions within the practice team	88	2.16 (0.81)	23	1.87 (0.81)	4	2.00 (0.82)	61	2.28 (0.80)
Group discussions	90	2.13 (0.74)	24	1.96 (0.81)	4	2.00 (0.82)	62	2.21 (0.70)
Presentations	88	2.17 (0.68)	24	2.04 (0.69)	4	2.00 (0.82)	60	2.23 (0.67)
Exchange with colleagues	89	2.13 (0.79)	24	2.08 (0.83)	4	1.75 (0.96)	61	2.18 (0.76)
Self-reflections	85	2.34 (0.73)	23	2.22 (0.85)	4	2.00 (0.82)	58	2.41 (0.68)
Overall project	98	2.55 (0.96)	25	2.32 (1.11)	4	2.50 (0.58)	69	2.64 (0.92)
Workshop 1 (leaders only)	22	1.95 (1.00)	22	1.95 (1.00)	–	–	–	–
Workshop 2	90	2.49 (0.94)	21	2.14 (1.01)	3	2.33 (0.58)	66	2.61 (0.91)

### Content analysis of practice leaders’ and practice assistants’ interviews

In addition to the quantitative evaluation, we conducted a total of seven structured interviews with 4 practice leaders and 3 practice assistants from 4 intervention practices. The main results of the content analysis are summarized here.

We identified eight common themes in the data: (1) strain due to the COVID-19 pandemic, (2) changes in working conditions and operational procedures, (3) project-related benefits, (4) changes in attitude, (5) persisting problems, (6) suggestions for improvement, (7) promoting factors for implementation, (8) barriers to implementation.

In all interviews, the COVID-19 pandemic was mentioned as the main barrier to implementation. New COVID-19-related (hygiene) regulations and documentation requirements, personal protective equipment procedures and patient management made the job even more challenging. During the 9-month implementation phase, this additional, pandemic-related workload profoundly impaired the implementation of strategies to achieve the practice goals. The frequently changing workplace requirements, new regulations, protective procedures for the practice team, increasing bureaucracy, and the pandemic-related additional workload with increasing hygiene requirements, coordination of appointments and changing administrative processes impaired the implementation of strategies to achieve the practice goals agreed upon in the intervention workshops.

The challenge of staying focussed on the goals due to the overall workload was reported as a persistent problem. Actual barriers to implementation included a shortage of staff and lack of time. Interviewees reported changes in some working conditions and operational procedures as presented in the workshops, resulting in improved communication within the team and with the patients. As a result of the skills training in the intervention workshops, participants mentioned a change in attitude towards patients, a questioning of current modes of operation and an increased awareness for change processes.

The results highlighted the following project-related promoting factors for implementation: the motivation for self-reflection, a regular exchange with other teams and the interaction with colleagues, the skills training, and practical demonstrations in the workshops. The interviews revealed some suggestions for improvement: practice assistants wanted less theoretical content, but more skills training. Also, encouragement for self-reflection in the workshops and more intensive on-site coaching were considered useful for future projects.

## Discussion

The innovative skills training-based IMPROVE*job* workshops were very well accepted by general practice leaders and their teams. Yet, the multimodal intervention had no effect on job satisfaction 9 months into the unforeseen COVID-19 pandemic which markedly impaired implementation processes. Several aspects need to be discussed to better understand the study results.

In medical education, mainly procedure-oriented leadership training is well established in the context of emergency, intensive care medicine and resuscitation, using standardized simulation exercises to train for the management of clearly defined clinical scenarios^26^. Focusing on interprofessional communication as a broader aim, surgical residents are trained by means of lectures, simulation exercises and scenarios^27^. As outlined in the reviews mentioned above, most current leadership training in medicine fails to address leadership as a broader topic and is not theory-based^3, 6, 7^. In human resource management research, a theory-based, long-term leadership development program with 25 leaders of a drug development corporation showed significant improvements in transformational leadership after five 2-day training sessions^28^. Based on such research from outside the field of medicine, Saravo et al. conducted a 4-week, on-the-job leadership training with skills training for medical residents addressing transformational and transactional aspects. In self- and observer ratings, the intervention group showed a significant improvement in both transformational and transactional leadership performance in the clinical setting^19^. Drawing on these successful experiences, the IMPROVE*job* leadership programme combined small group seminars with theoretical input on leadership, skills training and peer exchange to improve leadership among general practice leaders. This practice-oriented, theory- and skills training-based leadership program is a novelty that was widely accepted and rated well even by practice leaders with more than 20 years of experience as a physician.

With leadership as the most important predictor of job satisfaction^11^, the IMPROVE*job* study aimed to improve job satisfaction of general practice teams but was not successful in doing so 9 months into the pandemic. Several aspects might have played a role in this. First, our participants already showed a high level of job satisfaction at baseline, especially within the subgroups of practice leaders and employed physicians (COPSOQ 77.2 and 79.6; scale 0 to 100). The scores in our total sample were higher than the 2021 data of the COPSOQ databank with more than 200,000 participants from various occupational fields (74.19 vs. 63.1 of 100^11^). This is in line with prior research^29^ and makes interventions to improve job satisfaction more difficult. In contrast to other occupational groups^30^, our baseline data showed the interesting combination of high job satisfaction together with a high burden of chronic stress^25^. This finding of high chronic stress is in line with prior research^31^. Second, the early phase of the COVID-19 pandemic with its lockdowns and profound burden on primary care practices negatively impacted the 9-month implementation phase in two ways: Effective facilitator support was barely possible, and—most important as shown in the qualitative interviews—practices were extremely busy with COVID-19-related patient management, with no time for additional change processes geared at achieving their practice goal. Third, the profound impact of the COVID-19 pandemic on practices from both study arms likely outplayed any changes in the intervention group. This assumption is supported by the process evaluation and the finding that job satisfaction among leaders in the intervention group improved more than that among leaders from the control group, although significance was not reached when comparing the small subsamples. Fourth, change processes that rely on individual motivation and commitment^32, 33^ need time, especially if they involve a complex setting such as a practice. Leaders who were likely more motivated than practice assistants received a higher intervention dose as they participated in two workshops. This may have resulted in earlier mental change processes on behalf of the leaders, while the 9-month implementation phase likely was too short for changes of complex practice environments, especially within the scope of the pandemic. Supported by the theoretical framework of transfer training by Baldwin et al. several months are needed before subordinates may detect changes in leaders’ behaviors, with the exact mechanisms and time frames being unknown^28, 34^. Although transformational leadership is positively associated with a readiness to change^35^, high levels of occupational stress are negatively associated with attitudes and commitment towards change processes^36^, which played a major role in our practices in the face of the pandemic. Thus, the decrease in job satisfaction among practice assistants of our study might be attributable to a less transformational and more transactional leadership style to address the pandemic needs.

Our multilevel regression model on parameters that predict a change in job satisfaction identified higher age and a greater sense of community at follow-up as significant factors with relevance in both study arms. These findings are supported by Swedish research from successful change processes in intensive care units which identified five factors as relevant to integrating whole teams into team change processes: staff´s ownership of the change process; management has the role to initiate, coach and support the processes; team communication on values and norms; generous time allowance as the change processes take time; and room for re-evaluation^37^. A Polish study showed that good relations with trust among colleagues and to the supervisors are strongly associated with job satisfaction^38^, especially in the era of COVID-19 and the associated challenges.

### Strengths and limitations

The IMPROVE*job* study was a new approach to improve job satisfaction using a structured leadership intervention for the general practice setting. The cluster-randomised design including different practice types and whole practice teams was a strength of our study. In addition, we were able to draw on good data quality with a high level of completeness for the analyses, waiving the need for imputation. The newly developed IMPROVE*job* leadership program was well accepted, especially the moderated skills training including role-play with trained actors. The multi-professional composition of the research team and the range of contents presented allowed practices to individually select their focus based on their needs; however, the range might have been too broad but not deep enough for some practices. We developed the intervention in a participative approach with repeated input from practices and continuous input from a clinician scientist experienced in practice management. Practices with a very high psychological burden may not have participated in the study. The COVID-19 pandemic, which started between the baseline and follow-up assessments, impaired the study conduct, the implementation processes in practices and the participation in the follow-up data assessment.

## Conclusion

The newly developed IMPROVE*job* leadership program with its skills training was well accepted by participants, yet implementation was markedly impaired by the pandemic and the intervention did not improve job satisfaction. Based on the quantitative results, and supported by the qualitative interviews, further innovative approaches to enhance change processes in practices are needed to support the long-term well-being of practice leaders and practice assistants.

## Supplementary Information

Supplementary Information.

## Data Availability

There are no plans to grant access to the full protocol, participant-level dataset or statistical code as data contain potentially identifying information, but they are available from the corresponding author on reasonable request.
